# Psychometric evaluation of the Chinese version of the abbreviated technology anxiety scale among older patients with stroke: a translation and validation study

**DOI:** 10.3389/fpubh.2026.1823419

**Published:** 2026-05-08

**Authors:** Ru Zhang, Yao Sun, Mengxin Gan, Yujie Zhang, Chun Wen, Hongwen Ma

**Affiliations:** Department of Neurology, Tianjin Union Medical Center, The First Affiliated Hospital of Nankai University, Nankai University, Tianjin, China

**Keywords:** cross-cultural adaptation, reliability, stroke, technology anxiety, validation, validity

## Abstract

**Background:**

In the clinical care and rehabilitation of older patients with stroke, the application of technology has become increasingly prevalent, yet the issue of technology anxiety among this population has not received sufficient attention. Studies regarding technology anxiety in older patients with stroke are still rare in China due to the lack of reliable tools. This makes it difficult to accurately assess the actual level and core triggers of technology anxiety in older patients with stroke, thereby hindering the in-depth development of related studies. Information related to technology anxiety in Chinese older patients with stroke is still needed. Therefore, it is urgent to supplement research data on technology anxiety tailored to the characteristics of Chinese older patients with stroke, so as to provide data support and theoretical evidence for the formulation of subsequent intervention strategies.

**Objective:**

To translate the Abbreviated Technology Anxiety Scale (ATAS) into Chinese and test the reliability and validity of the Chinese version among older patients with stroke.

**Methods:**

Following Brislin’s translation principles, the original scale was subjected to forward translation, back-translation, and cultural adaptation to form the Chinese version of ATAS (C-ATAS). Using convenience sampling, 203 older patients with stroke from a tertiary hospital in Tianjin were recruited between November 2024 and April 2025 for a questionnaire survey to evaluate the reliability and validity of the C-ATAS.

**Results:**

The C-ATAS comprised 11 items and was a unidimensional scale. Exploratory factor analysis extracted one common factor, with factor loadings of items ranging from 0.747 to 0.964. The Cronbach’s alpha coefficient of the scale was 0.963, the split-half reliability was 0.962, and the test–retest reliability was 0.812. The content validity at the item level was 0.833–1.000, and the average content validity at the scale level was 0.970.

**Conclusion:**

The C-ATAS demonstrates good reliability and validity, making it an effective evaluation tool for technology anxiety in older patients with stroke. This study provides a reliable measurement tool for relevant clinical interventions and research on technology anxiety older patients with stroke. Future research should conduct more large-sample and multi-center studies to further verify the practicality and psychometric properties of the C-ATAS in other populations, as well as examine its psychometric properties across different cultural contexts.

## 1 Introduction

The aging of the population has become a global social development trend. The aging of China’s population is characterized by a large population base, fast development speed, and a deep degree of aging (1). Stroke, also known as apoplexy or cerebrovascular accident, is a syndrome of cerebral circulatory disorders caused by acute cerebrovascular diseases (2). Stroke is characterized by high incidence, high disability rate, high mortality rate, high recurrence rate, and high economic burden (3). With the advancement of population aging, the disease burden of stroke in China has shown an explosive growth trend (4, 5). In recent years, the incidence of stroke in China has been rising year by year, with the average age of onset in Chinese patients being 65 years old, and the older adults constituting the primary affected population, accounting for approximately 50.81% of the total cases (6). China is facing a severe aging situation with the health problems of the older adults becoming increasingly prominent, which poses enormous challenges to the medical and health system and nursing services (7).

Older patients with stroke have substantial medical needs, such as regular hospital visits to obtain oral medications (8). Leveraging its advantages of convenience, precision, and personalization, digital health technology helps older adults manage their health more effectively, improve their quality of life, and facilitate active aging (7, 9, 10). However, due to limitations such as physical functional decline and insufficient digital skills, many older adults often encounter difficulties in accessing and utilizing digital health technologies, and are prone to technological anxiety (11–13). Technology anxiety is an emotion oriented to the negative impacts of digital technologies such as mobile communication devices, artificial intelligence, and robots, leading to avoidance of information and communication technologies (14). Excessive technology anxiety may progress to technophobia, exerting adverse effects on older patients with stroke (15). Studies have shown that the technophobia of older patients with ischemic stroke cannot be ignored (8). Without timely intervention, excessive technology anxiety may develop into technophobia, significantly reducing patients’ adherence to digital rehabilitation, as reflected in operational avoidance, treatment interruption, and superficial engagement, thereby compromising rehabilitation outcomes (8). Therefore, employing technology anxiety assessment tools with satisfactory reliability and validity to effectively identify older stroke patients is of particular importance, holding significant clinical value for developing personalized digital rehabilitation strategies and improving long-term adherence.

However, studies regarding technology anxiety in older patients with stroke are still rare in China due to the lack of reliable tools. Information related to technology anxiety in Chinese older patients with stroke is still needed. The Abbreviated Technology Anxiety Scale (ATAS) has good reliability and validity and can be used as a tool to assess technology anxiety (15). Elrewany (16) validated and culturally adapted the Arabic version of the ATAS in five Arab countries, the results confirmed the reliability and validity of the Arabic ATAS as a self-report instrument to quantify technology anxiety among the general population. However, the ATAS has not been validated in Chinese older patients with stroke. Thus, it is necessary to rigorously cross-cultural adapt and validate the ATAS in Chinese older patients with stroke.

The aim of this study is to translate and evaluate the psychometric properties of the ATAS in Chinese older patients with stroke. Based on Wilson et al.’s results, a one-factor structure was hypothesized. Currently, only one technology anxiety scale has been validated among the older population in China, and there is no assessment tool for technology anxiety specifically designed for older inpatients, especially those with stroke (17). For older inpatients, a brief, validated, and reliable tool is needed to identify anxiety triggered by technology use in general. Therefore, Wilson et al. developed the 11-item Abbreviated Technology Anxiety Scale (ATAS), a self-reported scale measuring an individual’s level of technology anxiety. Thus, this study aims to achieve two objectives: first, translate and cross-culturally adapt the original ATAS into Chinese; second, evaluate its psychometric properties among Chinese older patients with stroke.

## 2 Methods

### 2.1 Translation and cross-cultural adaptation

This study obtained authorization by contacting the original authors via email, and then translated the ATAS into a Chinese version based on the modified Brislin model (18).

#### 2.1.1 Translation and back-translation of the scale

The research team was established (including 1 Doctor of Nursing, 3 Master of Nursing, 1 Master of Clinical Medicine, and 1 Bachelor of Nursing; among them, 1 Chief Nurse, 1 Associate Chief Nurse, 2 Associate Chief Physicians, 2 Senior Nurses, and 1 Nurse), and the Brislin model was used for localization (18).

(1) Literal translation: Two professionals were invited to translate, one of whom was a Doctor of Nursing (A1) proficient in professional English, and the other was a Master of Nursing (A2). The two translated versions were compared and integrated to form a comprehensive version I; (2) Back-translation: A Master of Nursing and an English teacher who had not been exposed to this questionnaire were asked to back-translate the comprehensive version I; (3) Translation review: The research team compared the back-translated version with the original scale, identified differences and made modifications. Finally, the final Chinese translation version and related documents were sent to the original author for confirmation to form the Chinese reviewed scale II.

#### 2.1.2 Cross-cultural adaptation of the scale

Six cross-cultural adaptation experts were included. The inclusion criteria for experts were: having a bachelor’s degree or above, a deputy senior professional title or above, and no less than 10 years of work experience. Finally, an expert panel was screened and confirmed, consisting of 1 nursing psychology expert, 2 clinical nursing experts, 2 nursing school professors, and 1 clinical medicine expert. The working years of the experts were as follows: in terms of educational background, there was 1 doctor, 3 masters, and 2 bachelors; in terms of professional titles, there were 2 senior professionals and 4 deputy senior professionals. The included experts evaluated the cultural relevance of the scale and proposed revision methods based on their personal practical experience, theoretical knowledge, domestic and foreign literature, and subjective feelings, finally forming the preliminary Chinese version of the ATAS.

#### 2.1.3 Pilot study

Convenience sampling was adopted, with the Department of Neurology of a tertiary Grade A hospital in Tianjin as the pilot survey site. A total of 30 older stroke patients who were hospitalized in October 2024 and met the inclusion and exclusion criteria were enrolled in this pilot study. The participants completed the preliminary Chinese version of the ATAS. For those who were unable to read the scale independently, the researchers read out the questions of each item verbally and recorded their responses. After the completion of the scale, the researchers inquired the older patients about the clarity and comprehensibility of each item content, investigated their experience during the completion process and their accurate understanding of the questionnaire content, and collected and documented the raised questions and suggestions. Following comprehensive analysis, discussion and revision by the research team, the initial Chinese version of the ATAS was finally developed.

### 2.2 Participants

This cross-sectional study was conducted from December 2024 to May 2025. Patients with stroke were recruited utilizing a convenience sample in the Department of Neurology and Rehabilitation of a tertiary first-class general hospital in Tianjin, China. The inclusion criteria for patients were as follows: (1) age ≥ 60 years; (2) clinically diagnosed with stroke; (3) conscious state; (4) native Chinese speaker; (5) stable vital signs; (6) voluntary participation and signed the informed consent form. The exclusion criteria were as follows: (1) patients had language disorders caused by focal lesions in the dominant hemisphere; (2) suffered from a major stress event.

The required sample size was calculated based on the principle that the sample size should be 5–10 times the number of items (19). The questionnaire consisted of 11 items, and considering a 10% invalid questionnaire rate, the estimated sample size ranged from 62 to 178 participants. Additionally, confirmatory factor analysis requires a sample size of at least 200 cases (20). Therefore, the final sample size determined for this study was 203 cases. This study was approved by the Ethics Committee of the affiliated hospital (Approval Number: Rapid Review No. B079 2026).

### 2.3 Instruments

#### 2.3.1 The general information questionnaire

The general information questionnaire was designed independently based on literature review and group discussions. The sociodemographic characteristics included participants’ gender, age, education level, marital status, monthly income (RMB), living status, and medical payment method. Disease-related variables included disease duration and comorbidity.

#### 2.3.2 The Chinese version of the abbreviated technology anxiety scale

The ATAS was developed by American scholars Wilson et al. (15) in 2023 to assess an individual’s level of technological anxiety. The C-ATAS consists of 11 items and is a unidimensional scale. All items adopt a 5-point Likert scale, with scores ranging from 1 to 5 corresponding to “strongly disagree” to “strongly agree” in sequence. The total score of the scale ranges from 11 to 55, with a higher score indicating a higher level of technological anxiety in individuals. The ATAS has good reliability and validity. Results of confirmatory factor analysis (CFA) in Wilson et al.’s study (15) show that the root mean square error of approximation (RMSEA) is 0.100, the comparative fit index (CFI) is 0.970, and the Tucker-Lewis index (TLI) is 0.970. Exploratory factor analysis (EFA) results show that a single factor exhibits an extremely significant eigenvalue. Standard correlation validity results show that ATAS scores are significantly negatively correlated with technological attitude, self-efficacy, and frequency of technology use (*p* < 0.001), and significantly positively correlated with anxiety (*p* < 0.001). The Cronbach’s alpha of the ATAS is 0.910. As a short-form scale, the ATAS is convenient for rapid assessment, and is suitable for research or low-risk scenarios (15).

### 2.4 Data collection process

Two trained and qualified researchers clarified the purpose and significance of this assessment to the research participants. After the voluntary participants signed the informed consent forms, they completed the collection of general demographic data and the assessment with the Chinese version of the ATAS in sequence. For the participants who could not fill in the scale independently due to advanced age, illiteracy or other reasons, the researchers assisted them in completing the filling via a word-by-word question-and-answer method. All research participants were recruited and finished the questionnaire survey during hospitalization. Two weeks later, 30 research participants were selected by convenience sampling to fill in the Chinese version of the ATAS for the second time (21).

### 2.5 Statistical analysis

The study data were independently entered into Excel by two researchers and cross-checked to ensure accuracy. SPSS 24.0 and SPSS Amos 24.0 software were used to analyze the data. Continuous variables were presented as frequencies and percentages. Descriptive statistics was expressed as mean ± standard deviation.

#### 2.5.1 Item analysis

The item analysis was conducted using the critical ratio (CR) test and the correlation coefficient method. The CR test was used to assess the discrimination of each item. The Chinese version of the ATAS scores of 203 older patients with stroke were sorted from highest to lowest. The top 27% of the total scores were designated as the high-score group, and the bottom 27% as the low-score group. An independent samples *t*-test was performed on the data of the two groups. If CR < 3, it indicated that the discrimination of each item was poor (22, 23). The correlation coefficient between the score of each item and the total scale score can reflect the homogeneity between the item and the total scale. A correlation coefficient *r* < 0.4 suggests a low correlation, and the item should be considered for deletion (24).

#### 2.5.2 Validity analysis

Validity evaluation included structural validity and content validity. The structural validity of the ATAS was evaluated using EFA. The Kaiser-Meyer-Olkin (KMO) values and Bartlett’s test sphericity were used for factor analysis. The cutoff point for item retention was the loading factor > 0.400 (22). Based on the results of EFA, CFA was further conducted. Goodness-of-fit indices were adopted to test the model fit, including *χ*^2^/*df*, RMSEA, GFI, AGFI, CFI, TLI and RMR. The acceptable criteria for model fit are defined as follows: *χ*^2^/*df* < 3, CFI > 0.8, GFI > 0.8, incremental fit index (IFI) > 0.8, TLI > 0.8, AGFI > 0.8, and RMSEA < 0.08. The ideal criteria for model fit are as follows: CFI > 0.9, GFI > 0.9, IFI > 0.9, TLI > 0.9, AGFI > 0.9, and RMSEA < 0.09 (20, 22). The content validity of the scale was evaluated using the expert evaluation method. Six experts in the fields of neurology and nursing were invited to assess the item content using a 4-point Likert scale. A score of 1 indicated irrelevant, 2 indicated weakly relevant, 3 indicated relatively relevant, and 4 indicated highly relevant (22). Comments were provided for items with disagreements. The item-level content validity index (I-CVI) and the average scale-level content validity index (S-CVI/Ave) were used for evaluation. The scale was considered to have good content validity when I-CVI ≥ 0.78 and S-CVI/Ave ≥ 0.9.

#### 2.5.3 Reliability analysis

Reliability testing includes internal consistency reliability, test–retest reliability, and split-half reliability. Internal consistency reliability was evaluated using the Cronbach’s alpha. A Cronbach’s alpha of 0.6–0.8 for the overall questionnaire indicates favorable internal consistency. The intraclass correlation coefficient (ICC) was used to evaluate the test–retest reliability of the scale, and an ICC value greater than 0.75 was considered acceptable (25). Split-half reliability was divided into two parts according to the sequential order of the items. The scores of the two parts were calculated separately, and the correlation coefficient between the two parts was compared. A correlation coefficient greater than 0.7 indicates favorable split-half reliability of the scale.

## 3 Results

### 3.1 Characteristics of participants

A total of 210 questionnaires were distributed, however, 7 invalid questionnaires were excluded due to more than 50% of the items being unanswered or the answers showing an obvious regular pattern. Finally, 203 (96.7%) participants were enrolled in the study and included in the final data analysis. The sociodemographic and clinical characteristics of the participants are summarized in Table 1. Among them, more than half 131 (64.5%) were male.

**Table 1:** Characteristics of 203 participants (*N* = 203).

Characteristics	*N*	%
Gender		
Male	131	64.5
Female	72	35.5
Age (years)		
60 ~ 70	101	49.8
71 ~ 79	81	39.9
≥80	21	10.3
Education level		
Primary school and below	43	21.2
Junior high school	67	33
Senior high school	67	33
College and above	26	12.8
Marital status		
Married	151	74.4
Other	52	25.6
Monthly income (RMB)		
<3,000	59	29.1
3,000 ~ 6,000	105	51.7
>6,000	39	19.2
Medical expense payment method		
Basic medical insurance for employees	149	73.4
Basic medical insurance for urban and rural residents	50	24.6
Self-funded	4	2
Living status		
Living alone	15	7.4
Living with spouse	175	86.2
Living with children	13	6.4
Disease duration (months)		
<3	153	75.4
3 ~ 12	42	20.7
>12	8	3.9
Number of comorbidities		
0	18	8.9
1	139	68.5
2	43	21.2
≥3	3	1.5

### 3.2 Cross-cultural adaptation

Based on the original version of the scale, this study carried out item language adaptation in accordance with the standardized procedures for cross-cultural adaptation to ensure the semantic accuracy, cultural compatibility and expression accessibility of the scale in Chinese research contexts.

Based on the results of expert consultation and cultural adaptation discussions, the research team decided to change “features” in item 2 “functions” to better fit the habits in Chinese culture. At the same time, we also suggest Item 5 from “Using technology makes me feel out of control” to “Using technology makes me lose control,” which streamlines redundant expressions while retaining the core semantic meaning of the original item. Item 9 was revised from “I am not familiar with technology” to “I am not very proficient in mastering technology,” which avoids the semantic ambiguity of the term “familiar,” makes the item expression more specific and targeted, and is more in line with the expression logic of Chinese speakers regarding experiences related to technological products.

### 3.3 Item analysis

The C-ATAS scores were divided into a high-score group (top 27%) and a low-score group (bottom 27%), and an independent samples *t*-test was conducted between the two groups. The results showed that when comparing the scores of each item between the high and low score groups, CR ranged from 12.375 to 47.706, and the differences were statistically significant (all *p* < 0.05), indicating that each item of the scale had good discriminative validity. Through Pearson correlation analysis, each item had a high degree of correlation with the total scale (*r* = 0.733 ~ 0.936, *p* < 0.001), so all items were retained.

### 3.4 Validity

#### 3.4.1 Structural validity

**Figure 1:**
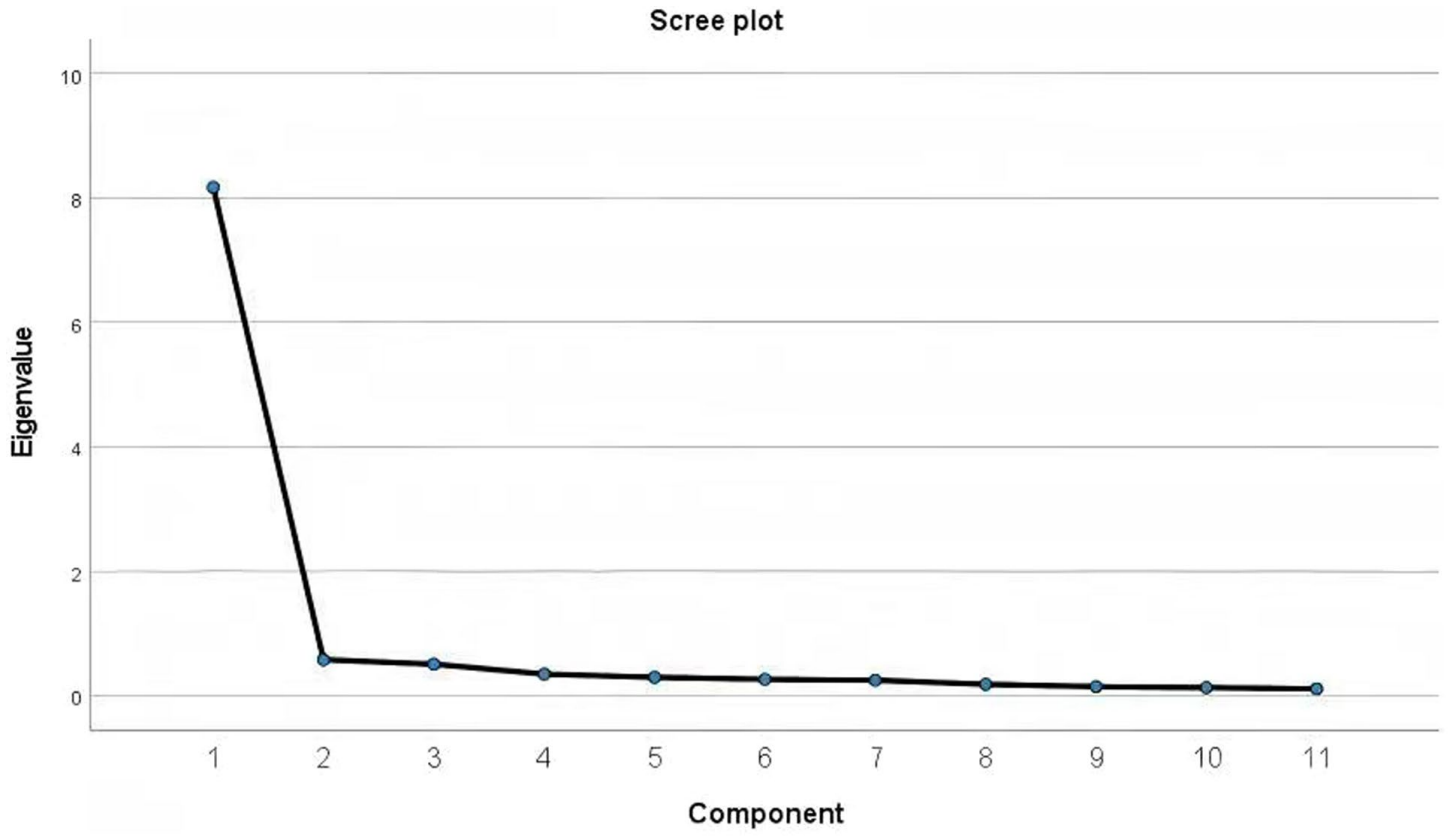
Scree plot of eigenvalues from the C-ATAS.

The value of KMO was 0.962 and Bartlett’s test of sphericity was significant (chi-square = 2351.064, *p* < 0.001), suggesting the factor analysis was feasible. The results of the EFA showed that all items were found to load on one factors for the full sample, and explained 74.83% of the variance (Figure 1; Table 2).

**Table 2:** Factor loading of EFA for C-ATAS.

Items	Factor loadings
1	0.839
2	0.964
3	0.952
4	0.924
5	0.862
6	0.952
7	0.927
8	0.881
9	0.906
10	0.747
11	0.918

**Figure 2:**
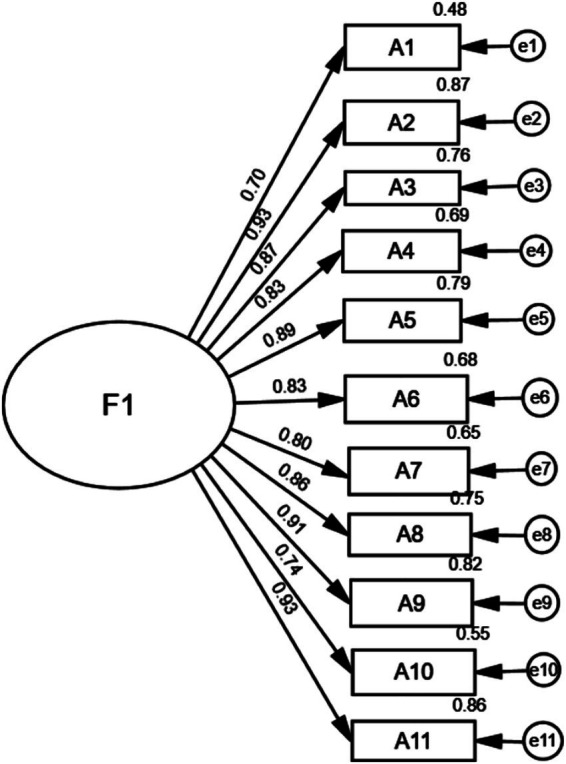
Standardized one-factor structural model of the C-ATAS.

The results of the CFA were as follows: *χ*^2^/*df* was 2.608, which was less than 3.000; the RMSEA was 0.042, which was less than 0.080; the CFI was 0.970, which was greater than 0.900; the GFI was 0.905, which was greater than 0.900; the TLI was 0.962, which was greater than 0.900; and the NFI was 0.952, which was greater than 0.900. The factor loadings of the scale items ranged from 0.696 to 0.933, all of which were greater than 0.40. The model fit generally met the criteria, indicating that the model was acceptable (Figure 2).

#### 3.4.2 Content validity

According to the evaluation results of the six experts, the I-CVI of the C-ATAS ranged from 0.833 to 1.000, and the S-CVI/Ave was 0.970, indicating favorable content validity.

### 3.5 Reliability

The Cronbach’s alpha coefficient of the C-ATAS was 0.963, which was greater than 0.800, indicating that the scale had good reliability and favorable internal consistency. The C-ATAS demonstrated excellent split-half reliability (*r* = 0.962) and good test–retest reliability [*I*CC = 0.812, *95% CI* (0.629, 0.908), *p* < 0.01], suggesting that the C-ATAS had high repeatability and satisfactory temporal stability.

## 4 Discussion

To the best of our knowledge, this is the first study in China to conduct standardized and rigorous translation, cross-cultural adaptation, and validity verification for the ATAS. The C-ATAS demonstrated sound scientificity and applicability among older patients with stroke.

### 4.1 Item analysis

The C-ATAS demonstrated good item discrimination. In accordance with the principles of item analysis, discriminability analysis was conducted on each item of the scale one by one to enhance the scale’s ability to distinguish and identify the technology anxiety levels of the research participants (26). Based on the Pearson correlation coefficient method, a Pearson correlation coefficient ranging from 0.30 to 0.80 between scale items and the total score is highly recommended (27). The correlation coefficients between the scores of each item and the total score of the C-ATAS were 0.733–0.936 (*p* < 0.05), indicating good internal homogeneity of the scale. This pattern is consistent with the original ATAS and its adapted versions, suggesting that the translation process did not compromise the internal consistency of the items.

### 4.2 The C-ATAS demonstrates good validity

Validity refers to the accuracy with which a measurement instrument reflects the concept being measured, and it is commonly evaluated by means of content validity, construct validity and other metrics (28, 29).

Content validity refers to the degree to which the items in a research instrument reflect the content to be measured (27). A scale is considered to have good content validity when its S-CVI ≥ 0.90 and I-CVI ≥ 0.70 (30). For the C-ATAS, the I-CVI ranged from 0.833 to 1.000 and the S-CVI was 0.970, indicating good content validity. The results confirm that the expert panel’s assessment of item-construct congruence was satisfactory and that the scale effectively covers the core connotations of technology anxiety at the content level.

In this study, a unidimensional factor analysis was conducted on the C-ATAS, which consists of 11 items, aiming to verify whether the scale can reflect the core concept of technology anxiety through a single dimension. This study examined the factor structure of the C-ATAS using EFA and CFA. The original ATAS (15) established a unidimensional factor structure based on a sample of U. S. college students. The EFA in this study extracted a single common factor, with a cumulative variance contribution rate comparable to that of the original version. The one-factor model demonstrated a good fit, which verified the factorial validity of the C-ATAS. The results of EFA showed that both the KMO test and Bartlett’s test of sphericity met the conditions for factor analysis, and one common factor was successfully extracted with a cumulative variance contribution rate of 74.83%. The factor loadings of all items were above 0.6, indicating that the unidimensional structure of the scale is reasonable and the results of the factor analysis are valid (31). As a classic method for testing structural validity, CFA can specifically verify the fit between a presupposed theoretical model and observed data, making up for the limitation of EFA that only focuses on data patterns while neglecting theoretical foundations (32). Based on the one-dimensional theoretical hypothesis of the original scale, this study constructed a one-dimensional CFA model and adopted the maximum likelihood estimation method to conduct fit analysis on the data, with the results interpreted focusing on two core dimensions: model fit and item factor loadings (33). In terms of model fit, three complementary types of indices—absolute fit indices, relative fit indices and parsimonious fit indices—were selected in this study to comprehensively judge the model fit effect and avoid biases caused by a single index. The results showed that the *χ*^2^/*df* value was 2.608, falling within the ideal range of 2.0–3.0, which indicated a good overall model fit; the RMSEA was 0.042, lower than the acceptable criterion of 0.080, suggesting that the approximate error of the model was within an acceptable range; the CFI, GFI, TLI and NFI were 0.970, 0.905, 0.962, and 0.952, respectively, all exceeding the critical value of 0.900. These findings demonstrated that the target model was significantly improved compared with the baseline independent model and could effectively explain the data variation. The comprehensive performance of the above fit indices indicated that the one-dimensional structure of the C-ATAS was consistent with the theoretical conception of the original scale, confirming that the Sinicization process did not disrupt the core dimensional structure of the scale, and that technology anxiety also manifests as a unidimensional construct among older stroke patients.

Regarding item factor loadings, the standardized factor loading coefficients of all items ranged from 0.696 to 0.933, all above the critical value of 0.40 and reaching a significant level of *p* < 0.001. This indicated that each item could effectively reflect the connotation of the one-dimensional latent variable, with no invalid items or items with weak loadings identified (34). The error terms of all items were positive and within a reasonable range, with no negative error observed, which suggested that the measurement error of each item was small, the measurement reliability was high, and the redundancy among items was low. All items could independently provide valid information for the latent variable, which further supported the rationality of the one-dimensional structure of the scale and verified the accuracy of item translation and expression adjustment in the Sinicization process. That is, the process not only ensured linguistic fluency and local adaptation but also completely retained the core connotation of the target construct measured by the original items, without causing deviations in the measurement direction or focus of the items due to cultural adaptation.

### 4.3 The C-ATAS demonstrates good reliability

Reliability refers to the consistency of measurement results under repeated testing or different conditions, reflecting the ability of a measurement instrument to eliminate random errors (35, 36). The results of this study showed that the Cronbach’s alpha coefficient of the C-ATAS was 0.963 (> 0.800), which was similar to that of the original scale, indicating that the scale had good reliability and favorable internal consistency. The split-half reliability of the scale was 0.962 (> 0.700). The test–retest reliability of the C-ATAS was 0.812, suggesting that the scale was reliable and possessed satisfactory temporal stability. Notably, the Cronbach’s alpha of the C-ATAS was higher than that of the original ATAS (Cronbach’s alpha = 0.910) and similar to that of the Arabic version (Cronbach’s alpha = 0.960). This phenomenon can be interpreted from two perspectives. First, the high alpha value may reflect sample homogeneity. The population of older stroke patients is relatively homogeneous in terms of age, disease experience, and level of exposure to digital devices, which may reduce random variation in item responses and thereby inflate reliability estimates. Second, unlike the college student samples used in other ATAS adaptation studies, the present study focused on older stroke patients. These two populations differ substantially in age, cognitive level, and digital device experience, which may lead to different manifestations of technology anxiety and different patterns of item responding, thereby influencing reliability estimates. Differences in the manifestation of technology anxiety, degree of variability, and item comprehension across these populations may also influence reliability estimates (37).

In this study, the Brislin translation model was adopted to conduct translation, back-translation and cross-cultural adaptation of the C-ATAS, and the C-ATAS with 11 items was finally developed. The results of this study indicate that the C-ATAS has favorable reliability and validity. From the perspective of clinical practice, the C-ATAS has a moderate number of items, requires a short completion time, and features simple item expressions that do not impose a burden on patients. It is suitable for use by older patients and presents good accessibility.

### 4.4 The C-ATAS scale demonstrates good scientific rigor and practical applicability

The results of this study indicated that the C-ATAS has satisfactory reliability and validity with brief, clear items and short completion time, ensuring good clinical feasibility. From the perspective of clinical practice, the scale features brief, clear and easy-to-understand item descriptions with a moderate number of items and a short completion time, which imposes no burden on patients and thus exhibits good feasibility (38). From a practical standpoint, the C-ATAS can assist clinical medical staff in accurately assessing the level of technology anxiety in older patients with stroke, thereby providing a reference and guidance for formulating multi-faceted and personalized intervention plans and facilitating the achievement of healthy aging. Conducting technology anxiety assessment helps identify various problems encountered by older patients with stroke in the process of using digital devices, such as fear of operation, excessive concerns about privacy and security, and anxiety caused by excessive cognitive load. On this basis, targeted and stratified interventions can be implemented to improve long-term adherence to digital rehabilitation. For example, simplified user interfaces, human assistance, and gradual technology adaptation can be provided to patients with high anxiety during the early screening stage (39). This can avoid the negative effects caused by excessive feedback frequency. Such negative effects do not simply stem from cognitive load, but are essentially caused by technology overload (40). Future work will center on older stroke patients, conduct co-design research to explore how their technology anxiety spectrum, information presentation preferences, task difficulty tolerance, and other factors influence tool design, and combine usability and cognitive load evaluation to develop age-specific design optimization strategies (41).

### 4.5 Limitations

This study has several limitations. First, both EFA and CFA were conducted on the same sample without sample splitting or independent cross-validation, which may risk inflating model fit indices. However, given that this was a preliminary validation study designed to initially explore and verify the factor structure of the C-ATAS, splitting the sample (e.g., 100 for EFA and 103 for CFA) would have resulted in insufficient statistical power in both subsamples, making it difficult to obtain stable factor solutions. Thus, the present study provides a foundation for subsequent large-scale validation. Future studies should validate the C-ATAS factor structure using an independent sample or cross-validation approach to confirm the generalizability of the current findings. Second, participants were recruited from a single department at one tertiary Grade A hospital in Tianjin, with a limited sample size. Future research should expand to multiple centers and geographic regions and enroll larger samples to further evaluate the applicability of the C-ATAS among older stroke patients in China. Longitudinal studies are also warranted to examine the predictive utility of the C-ATAS.

## 5 Conclusion

The C-ATAS demonstrates good reliability and validity, making it a suitable instrument for effectively assessing the level of technology anxiety among older patients with stroke in the Chinese cultural context. However, this study is a single-center investigation with a relatively limited sample size. In future research, more large-sample and multi-center studies can be conducted to further verify the practicality of the C-ATAS and its psychometric properties in other populations.

## Data Availability

The raw data supporting the conclusions of this article will be made available by the authors, without undue reservation.
